# Physiologic perfusion monitoring methods during endovascular revascularization for atherosclerotic peripheral arterial disease: protocol for a systematic review

**DOI:** 10.1186/s13643-020-01357-y

**Published:** 2020-05-08

**Authors:** Mark Rockley, Prasad Jetty, George Wells

**Affiliations:** 1 Division of Vascular and Endovascular Surgery, Department of Surgery, University of Ottawa, The Ottawa Hospital-Civic Campus, K1Y4E9, Ottawa, Canada; 2 School of Epidemiology and Public Health, Cardiovascular Research Methods Centre, University of Ottawa Heart Institute, K1Y4W7, Ottawa, Canada

**Keywords:** Angioplasty, Peripheral arterial disease, Perfusion, Intraoperative monitoring

## Abstract

**Background:**

Endovascular therapy is a fundamental treatment for peripheral arterial disease. However, the success rate of endovascular therapy remains poor, as a third of patients with critical limb ischemia ultimately require a major amputation for gangrene despite endovascular treatment. This failure rate has prompted investigation into methods of determining physiologic procedural success before and after treatment, before clinically apparent outcomes occur such as gangrene. The aim of this systematic review is to evaluate if in patients undergoing endovascular surgery for lower extremity atherosclerotic peripheral arterial disease, do changes in physiologic measures of perfusion during surgery correlate with clinical outcomes.

**Methods:**

We registered and designed a study protocol for a systematic review. Literature searches will be conducted in MEDLINE, EMBASE, and CENTRAL (from January 1977 onwards). Grey literature will be identified through OpenGrey and clinical trial registries, and supplemented by citation searches. We will include randomized controlled trials, quasi-experimental trials, and observational (cohort, case-control) studies conducted in human adults (age 18 or older) who received elective arterial angioplasty for atherosclerotic peripheral vascular disease. The primary outcome of interest will be major adverse limb events. Two investigators will independently screen all citation, full-text articles, and abstract data. The study quality (risk of bias) will be appraised appropriate tools. Data analysis and synthesis will be qualitative; no meta-analysis is planned, as the anticipated homogeneity of measurement and outcome reporting standardization is low.

**Discussion:**

The treatment of peripheral arterial disease is unique in that the tissue of the ischemic leg is easily accessible for direct monitoring during procedures. This is contrasted with cardiac and neurologic monitoring during cardiac and cerebral procedures, where indirect or invasive measures are required to monitor organ perfusion. Currently synthesized evidence describing limb perfusion focuses on static states of ischemia, and does not evaluate the value of change in perfusion measurement as an indicator of endovascular treatment success. These methods could potentially be applied to optimize procedural outcomes by guiding perfusion-based decision-making during surgery.

**Systematic review registration:**

PROSPERO CRD42019138192

## Background

### Rationale

Peripheral vascular disease (PVD) is a condition cause by arterial blockages causing inadequate blood flow, resulting in pain and gangrene of the legs. The prevalence of PVD in the North American general population over 50 years of age is estimated at 17.4%, and is rising in association with the increasing prevalence of diabetes [1]. Bypass surgery is typically reserved for patients with severe forms of PVD, and minimally invasive options of angioplasty (“endovascular surgery”) are emerging as the treatment of choice for most patients with PVD. Angioplasty is the foundational treatment of endovascular therapy, which may be augmented by treatments such as stenting and atherectomy. Unfortunately, the 2-year patency of balloon angioplasty for PVD is poor, reported between 50–80%, depending on lesion location and characteristics [2]. The 1-year amputation rate despite endovascular revascularization has been reported as high as 32% in patients with lower limb critical limb ischemia [3]. This has prompted investigation into the predictors of failure, and potential solutions to optimize the success rate of revascularization. One proposed method is to evaluate the physiologic improvement in limb perfusion intraoperatively, to provide the operator with an opportunity to evaluate the procedural success and potentially guide intraoperative decision-making.

One of the most important predictors of clinical success following endovascular surgery for PVD is the post-procedure ankle-brachial index (ABI) [4]. This measurement is performed by applying a blood pressure cuff at the level of the lower leg (“Ankle Pressure”) and the arm. The ABI is a ratio of the blood pressure at the ankle when compared with the arm. Similarly, a smaller cuff around the great toe can determine the absolute toe pressure, which can also be used to calculate the toe-brachial index (TBI). The change in ABI following endovascular surgery can be detected a day after the procedure, and remains stable throughout the month following the procedure [5]. Several other postoperative markers of limb perfusion have also been investigated. Magnetic resonance arterial spin labeling correlates with postoperative ABI and clinical outcomes [6]. Furthermore, some authors have investigated markers of limb perfusion during surgery, finding correlation between postoperative ABI and intraoperative 2-dimensional perfusion angiography [7] and indocyanine green intra-arterial injection [8]. Other methods such as laser doppler [9], near-infrared spectroscopy [10, 11], transcutaneous oxygen saturation [12], and micro-oxygen sensors [13] have also been evaluated. While these methods have been established as markers of perfusion in the outpatient setting, their role in guiding intraoperative decision-making is unclear.

The potential of physiologic measures to predict clinical outcomes after endovascular revascularization presents several opportunities. The current practice of waiting until the postoperative period to measure the limb pressure after surgery may miss opportunities to guide intraoperative decision-making. While angiogram is currently the primary form of intraoperative feedback, conventional angiogram provides only anatomic feedback, which may not correlate with physiologic perfusion of blood due to microvascular disease and diffuse disease.

### Objectives

The aim of this systematic review is to evaluate if in patients undergoing endovascular surgery for lower extremity atherosclerotic peripheral arterial disease, do changes in physiologic measures of limb perfusion during surgery correlate with clinical outcomes. Physiologic measures include non-invasive and invasive arterial pressure measurements, transcutaneous oxygen measurement, infrared spectroscopy, laser doppler flowmetry, and angiogram perfusion calculations.

Secondary questions that will be addressed by this review will investigate the correlation of intraoperative physiologic measures with non-clinical postoperative outcomes such as radiographic patency and hemodynamic outcomes.

## Methods

### Eligibility criteria

#### Study designs

We will include randomized controlled trials (RCT) and quasi-experimental trials, non-randomized controlled trials, cluster trials, interrupted time series studies, controlled before-after studies (CBA), prospective or retrospective cohort studies, and case-control studies. Case series less than 5 participants and case reports will be excluded.

#### Participants

We will include studies examining human adults (age 18 or older) who received elective arterial angioplasty for atherosclerotic peripheral vascular disease. The angioplasty must be the primary purpose of the intervention, and not be performed concurrently with a hybrid open vascular procedure on an in-line flow artery. The intervention must be performed on lower extremity peripheral arteries, ranging from the infrarenal aorta proximally to the toes distally. The balloons may be drug-coated or lined with cutting ribs, and alternate adjunctive endovascular procedures stent placement, orbital atherectomy, laser atherectomy, rotational atherectomy, or directional atherectomy. We will exclude venous angioplasty, arteriovenous fistula angioplasty, and studies examining emergency settings.

#### Intervention and comparators

The intervention of interest is intraoperative physiologic measurement of limb perfusion. Examples of established physiologic measurements of perfusion include non-invasive and invasive arterial pressure measurements, transcutaneous oxygen measurement, infrared spectroscopy, laser doppler flowmetry, and angiogram perfusion. Any further methods of physiologic measurement which are currently unknown to the authors and are discovered during the review will be considered individually. They will be included in the review if they meet our definition of intraoperative physiologic limb perfusion measurement, dynamic measures of blood flow within the target limb performed during surgery. Strictly anatomic measurements which are available on standard angiogram, such as residual stenosis or patency, will be excluded.

#### Outcomes

The primary outcome of interest will be major adverse limb events (MALE). MALE is defined as a composite outcome of clinically driven target limb reintervention, and major amputation proximal to the ankle [14].

Secondary clinical outcomes include amputation, reintervention, mortality, and clinical symptomatic improvement based on the Rutherford’s classification of peripheral vascular disease [15]. Secondary non-clinical outcomes include non-invasive limb hemodynamic results, and radiographic results of vessel patency and stenosis.

#### Timing

Studies will be selected for inclusion if they report intraoperative monitoring results in addition to follow up outcomes reported at least 30 days after the index surgery, categorized into clinical, radiographic, and hemodynamic outcomes.

#### Setting

There are no restrictions regarding setting of the study.

#### Language

We will include all language studies, with a list of titles requiring translation into English included in an appendix.

### Information sources

A literature search strategy using medical subject headings and text words has been developed. We will search MEDLINE (OVID interface), EMBASE (OVID interface), and the Cochrane Central Register of Controlled Trials (Wiley interface).

To ensure capture of all relevant trials, all selected studies will also undergo ancestry search, in addition to citation search using SCOPUS. OpenGrey will be interrogated for unpublished relevant literature.

### Search strategy

Both qualitative and quantitative studies will be collected. All searches will be limited by date of publication (January 1977–onwards). The initial year of 1977 has been chosen as the first in-human use of angioplasty was performed that year. No language limit will be placed on the search. The search strategy and syntax have been guided by a Health Sciences librarian with systematic review experience. The MEDLINE search syntax will be adapted to accommodate the remaining database searches. Please see Appendix for a complete search syntax used for the MEDLINE search. The search syntax is intentionally broad to include any potentially relevant methods of perfusion measurement. Of note, the PROSPERO database has been searched, and no ongoing or recently completed systematic review on this topic has been performed.

### Study records

#### Data management

Literature search results will be aggregated in EndNote, including where duplicate articles will be removed. The results will then be uploaded to the Distiller SR software, which will facilitate collaboration among all reviewers.

The two screening authors will independently screen titles and abstracts resulting from the combined search of all selected databases. The full text of an article will be obtained for any articles that appear to meet eligibility criteria, at which point the full text will be screened and confirmation of article inclusion will be made. Any reasons for exclusion following full text screening will be explicitly documented and listed in an appendix.

Once both reviewers have created a complete list of eligible articles, the lists will be compared. Discrepancies in article selection will be addressed with discussion with a third-party author experienced in systematic review conduct. No authors will be blinded to journal titles, study authors, or study location of origin.

#### Data collection process

A standardized form created in Distiller SR will be used as the data collection method. Both reviewers will have a separate form for each article, which will be compared for consistency after data collection has completed. Any discrepancy will be addressed with discussion with a third-party author experienced in systematic review conduct. Study authors will not be contacted to resolve unclear or inadequate reporting of data.

### Data items

The trial design, setting, and any accessory measures such as observer or operator blinding will be assessed. We will collect patient population information including level of endovascular procedure, claudication versus critical limb ischemia, use of vasodilators such as cilostazol or pentoxifylline, and comorbidity data. We will extract perfusion monitoring information including method of perfusion monitoring, timing of monitoring, and any objective serial outcomes, their confidence measures, variability, and inter-rater reliability. We will also note any author comments on feasibility or obstacles in using the method. Extracted outcomes will be guided by the definitions below.

### Outcomes and prioritization

#### Primary outcome

The primary outcome of interest will be major adverse limb events (MALE). MALE is defined as a composite outcome of clinically driven target limb reintervention, and major amputation proximal to the ankle [14].

#### Secondary outcomes

Secondary clinical outcomes include the following:

- Amputation
  - Minor amputation (toe(s) or foot)
  - Major amputation (above the ankle)
  - Amputation-free survival
- Reintervention
  - Target:
    - Lesion
    - Vessel
    - Limb
  - Endovascular
  - Bypass
  - Thrombectomy
  - Thrombolysis
- Mortality
- Improvement in Rutherford’s classification of peripheral vascular disease [15]

Secondary non-clinical outcomes include the following:

- Postoperative hemodynamic
  - Ankle pressure
  - Ankle-brachial index (ABI)
  - Toe pressure
  - Toe-brachial index (TBI)
- Postoperative radiographic
  - Target vessel patency
    - Primary (absence of target vessel occlusion or restenosis > 50%)
    - Primary assisted (patency requiring assistance of subsequent procedure to maintain patency of target vessel)
    - Secondary (patency requiring assistance of subsequent procedure to restore patency of target vessel)

### Risk of bias of individual studies

To assess individual studies for potential risk of bias, we will collect information guided by the Cochrane Collaboration Risk of Bias 2.0 tool [16]. In summary, this includes sequence generation, allocation concealment, blinding, incomplete outcome data, and selective outcome reporting. For each category, each study will be determined to be at either low or high risk. Alternately, if the report includes insufficient information to determine the level of risk, the category will be labeled as unclear. The quality of cohort studies will be assessed by the Newcastle-Ottawa Scale [17], and the quality of case series will be assessed by the Institute for Health Economics Quality Appraisal Checklist [18, 19]. Determination of the level of bias will be made by the two reviewers independently, and compared following complete assessment of all studies. Any discrepancy will be addressed with discussion with a third-party author experienced in systematic review conduct. The resulting risk of bias for each study, in each category, will be graphically represented by the RevMan software.

### Data synthesis

#### Quantitative synthesis

Due to the anticipated heterogeneity of reports, including the methods of perfusion monitoring, clinical outcomes, and type of endovascular surgery, we will not perform quantitative data synthesis.

#### Qualitative synthesis

We will summarize the described methods of intraoperative perfusion measurement. Specifically, we will describe the reported association of these methods with postoperative outcomes. We will comment on the reported variations in methods, and assess the volume of published experience in utilizing each method. We will also comment on any practical information gleaned from the review. This will include comments on feasibility, reliability, and accessibility. We will also describe any reported patient characteristics that may affect the method’s usability. Where there is missing data, we will contact the authors of the original study to obtain complete data where possible.

#### A priori subgroup analyses

We will stratify our qualitative analysis into the three postoperative outcome categories: clinical, hemodynamic, and radiographic. Where possible, we will stratify the qualitative analysis by vascular level of intervention (aortoiliac, femoropopliteal, or tibial), symptom status (claudication or critical limb ischemia), diabetic status, and use of peripheral vasodilators such as cilostazol or pentoxifylline.

#### Meta-bias

As there is no planned quantitative data synthesis, we will not perform statistical analysis of meta-bias. We will however comment on the risk of bias of each study, as well as qualitatively describe.

#### Confidence in cumulative estimate

There will not be a cumulative estimate produced by this study, and therefore there will be no assessment of confidence. However, the generalizability of the findings to the PVD patient population will be qualitatively judged.

## Discussion

In summary, the success of endovascular therapy for PVD is relatively poor despite technological advancements, resulting in a high reintervention rate. A component of this challenge may be inadequate revascularization during the initial procedure, despite a reassuring anatomic result on angiogram. Physiologic measures of limb perfusion may provide insight into which patients are incompletely revascularized during the initial procedure. If intraoperative feedback of limb perfusion is predictive of outcomes, these methods could be used to guide intraoperative decision-making and ultimately improve the success of endovascular revascularization.

This review may be limited by the anticipated lack of standardized reporting or high-quality evidence including prospective randomized trials. In response, we do not plan to perform any meta-analyses. Qualitative synthesis alone will summarize existing literature, but is unlikely to directly influence current practice by itself. This review is still warranted however, as even lower quality studies may provide useful insights to guide more robust future investigation, which may help to inform future practice.

If we encounter the need for a protocol amendment, the change must be approved by all study members. The protocol change will be immediately updated in the PROSPERO registration, and the change will be explicitly described in the methods section of the final manuscript.

## Data Availability

The datasets generated and analyzed during the current study will be available from the corresponding author on reasonable request.

## Appendix

### Appendix: Proposed search syntax for MEDLINE, using OVID interface

Peripheral vascular disease

1. Peripheral Vascular Diseases/
2. GANGRENE/
3. INTERMITTENT CLAUDICATION/
4. Peripheral Arterial Disease/
5. PVD.tw.
6. peripheral vascular disease*.tw.
7. Peripheral angiopath*.tw
8. peripheral arterial disease*.tw.
9. gangrene*.tw.
10. claudica*.tw.
11. critical limb ischemia*.tw.
12. (1 to 11, OR)

Endovascular revascularization

13. Angioplasty/
14. ENDOVASCULAR PROCEDURES/
15. Stents/
16. angioplast*.tw.
17. endovascular*.tw.
18. stent*.tw.
19. atherectom*.tw.
20. (Endoluminal adj3 repair*).tw.
21. (13 to 20, OR)

Intraoperative monitoring

22. Monitoring, Intraoperative/
23. (intraoperative adj3 monitor*).tw.
24. (perfusion adj3 angio*).tw.
25. Ankle Brachial Index/
26. Arterial Pressure/
27. ankle brachial.tw.
28. (ankle adj3 pressur*).tw.
29. toe brachial.tw.
30. (toe adj3 pressur*).tw.
31. ABI.tw.
32. TBI.tw.
33. Blood Gas Monitoring, Transcutaneous/
34. (transcutaneous adj2 oxygen pressur*).tw.
35. tcpo2.tw.
36. Spectrophotometry, Infrared/ or Spectroscopy, Near-Infrared/
37. infrared spectro*.tw.
38. correlation spectroscop*.tw.
39. Pulse volume*.tw.
40. PVR.tw.
41. Tomography, Optical Coherence/
42. (optic* adj2 coheren*).tw.
43. Laser-Doppler Flowmetry/
44. (laser adj2 doppler).tw.
45. (22 to 44, OR)
46. (12 AND 21 AND 45)

## Abbreviations

PVD: Peripheral vascular disease
MALE: Major adverse limb events
ABI: Ankle-brachial index
TBI: Toe-brachial index
